# Cohort Changes in Cognitive Function Among Mexican Older Adults from 2001 to 2021

**DOI:** 10.1093/geront/gnaf143

**Published:** 2025-05-28

**Authors:** Julián Ponce, Hiram Beltrán-Sánchez

**Affiliations:** Department of Community Health Sciences, Fielding School of Public Health, University of California Los Angeles (UCLA), Los Angeles, California, USA; Department of Community Health Sciences, Fielding School of Public Health, University of California Los Angeles (UCLA), Los Angeles, California, USA; UCLA California Center for Population Research, University of California Los Angeles (UCLA), Los Angeles, California, USA

**Keywords:** Cognitive decline, Mexico, Multimorbidity

## Abstract

**Background and Objectives:**

Multimorbidity (2+ chronic conditions) associated with faster cognitive decline among older adults, yet longitudinal evidence from low- and middle-income countries, including Mexico, remains limited. This study examines cohort differences in the annual rate of cognitive decline, measured by global cognitive function scores (GCFS), and tests whether the association between multimorbidity and cognitive decline differs between two cohorts aged 50–60 in 2001 and 2012.

**Research Design and Methods:**

We assess two 10-year birth cohorts (Cohort 1: born 1941–1951, *n* = 5,345 Cohort 2: born 1952–1962, *n* = 4,378), at 3 time points (Cohort 1: 2001, 2003, and 2012; Cohort 2: 2012, 2015, 2021), at ages 50–60 at baseline. We examine cohort differences in average annual GCFS changes by fitting growth curve models incorporating random intercepts and slopes.

**Results:**

Two key findings emerged. First, the earlier cohort (Cohort 1, 2001), experienced a faster average annual rate of decline in GCFS than the recent cohort (Cohort 2, 2012). Second, the link between multimorbidity and cognitive decline did not significantly differ between cohorts net of possible confounders.

**Discussion and Implications:**

Our findings advance our understanding of cohort differences in cognitive decline and how the influence of multimorbidity on cognitive decline has evolved in Mexico. The slower rate of decline among the recent cohort suggests potential improvements in cognitive reserve due to educational improvements. Improvements in healthcare access over the past decades may have mitigated the negative consequences of multimorbidity on cognitive decline, potentially explaining the absence of cohort differences.

## Background and Objectives

Globally, multimorbidity (co-occurrence of ≥2 chronic conditions) has emerged as a significant public health challenge, reflecting systemic health inequalities across demographic groups and geographic settings (Chowdhury et al., 2023; Ho et al., 2022; Kone et al., 2021; Mohamud et al., 2023; Rojas-Huerta et al., 2022; Siah et al., 2022). Estimates suggest a global prevalence ranging from 38.6% to 42.4%, with certain groups disproportionately affected, including women, older adults, and those of lower socioeconomic status (SES; Chowdhury et al., 2023; Ho et al., 2022; Kone et al., 2021; Mohamud et al., 2023; Rojas-Huerta et al., 2022; Siah et al., 2022). Moreover, multimorbidity prevalence varies across regions, with South America estimated to have the highest prevalence (45.7%), followed by North America (43.1%; Chowdhury et al., 2023). Even in parts of Europe with low prevalence, multimorbidity has increased, with some studies indicating a 2.5 percentage point rise over the last decade (Siah et al., 2022; Souza et al., 2021). Importantly, many low- and middle-income countries (LMICs) now face multimorbidity rates rivaling high-income countries, a trend fueled by rapid population aging and persistent socioeconomic inequalities (Chowdhury et al., 2023).

The high burden of multimorbidity, particularly from cardiometabolic conditions like hypertension, diabetes, and heart disease, falls disproportionately on socioeconomically disadvantaged groups (Dove et al., 2023; Fajardo Dolci et al., 2023; Jin et al., 2023; Khondoker et al., 2023; Singer et al., 2019; Souza et al., 2021). This disparity is largely driven by educational and wealth inequalities, with individuals of low SES facing the greatest burden of multimorbidity (Buffel du Vaure et al., 2016; Pathirana & Jackson, 2018; Pearson-Stuttard et al., 2019; Siah et al., 2022). For example, individuals with lower education face 64% higher odds of multimorbidity (Pathirana & Jackson, 2018), while those with less wealth have a 47% greater risk (Singer et al., 2019). These socioeconomic disparities produce divergent health trajectories across the life course, with lower-SES groups and regions with larger economic disparities experiencing more multimorbidity and its complications (Dannefer, 2003; Pathirana & Jackson, 2018). These divergent health trajectories can be extended to birth cohorts that share distinct socioeconomic histories, which generate variation in vulnerability to multimorbidity and its health consequences. As a result, broader social and epidemiological shifts shape generational differences in aging outcomes, influencing the prevalence of multimorbidity and cognitive function.

Multimorbidity and in particular, conditions related to cardiometabolic functioning are highly predictive of cognitive function and dementia in older adults (Dove et al., 2023; Jin et al., 2023; Khondoker et al., 2023; Luo et al., 2023; Posis et al., 2024; Wei et al., 2020; Xing et al., 2024; Zhou et al., 2024). For example, a Swedish study found cardiometabolic multimorbidity to accelerate the onset of cognitive impairment no dementia by 2.3 years and dementia by 1.8 years among adults aged 60 and over (Dove et al., 2023) and in the U.K., people with multimorbidity had a 102% increased risk of developing dementia (Khondoker et al., 2023). Some evidence suggests that cardiometabolic conditions may drive cognitive decline through various biological pathways including the cardiovascular system (e.g., reduced blood flow, vascular lesions), inflammation, and structural changes to the brain, especially among older adults who already exhibit reduced blood flow (de la Torre, 2012; Jensen et al., 2023). These mechanisms may be particularly important in LMICs, where undiagnosed cardiovascular conditions interact with socioeconomic disparities to amplify cognitive risks across successive aging cohorts (Bloom & Luca, 2016; de la Torre, 2012; Jensen et al., 2023). Such variations ultimately reflect fundamental differences in how structural contexts shape multimorbidity’s association with cognition, differences that become apparent when comparing outcomes across nations and age groups.

The detrimental link between multimorbidity and cognitive decline is further complicated by context-dependent variations, with longitudinal studies revealing striking cross-national differences in its severity and timing (Luo et al., 2023; Posis et al., 2024; Wei et al., 2020; Xing et al., 2024; Zhou et al., 2024). For instance, middle-aged Chinese adults (40+) with cardiometabolic multimorbidity exhibited significant cognitive decline (*β* = − 0.23) over seven years (Zhou et al., 2024), suggesting the detrimental association may emerge earlier than previously assumed. In contrast, U.S. studies of older adults (60+) reported a more moderate decline (*β* = − 0.20; Posis et al., 2024), highlighting disparities in magnitude across age groups by country. Yet most evidence on this topic stems from high-income countries with strong healthcare systems, where older populations benefit from better disease management (Luo et al., 2023; Posis et al., 2024; Wei et al., 2020; Xing et al., 2024; Zhou et al., 2024). This leaves a critical gap in understanding LMICs, where fragmented healthcare and accelerated aging may exacerbate cognitive decline, potentially at younger ages and with greater severity than currently documented (Bloom & Luca, 2016).

These context-dependent patterns of multimorbidity’s association with cognition become especially critical in LMICs, where rapid population aging has outpaced healthcare improvements (Kalaria et al., 2024; UN, 2020). By 2050, two-thirds of the world’s older adults (60+) will reside in LMICs (UN, 2020), populations already facing disproportionate burdens. For example, multimorbidity is already widespread in LMICs, affecting 32.1% of adults over 30 across 24 LMICs and rising to 51% of those aged 60 and older globally (Chowdhury et al., 2023). Notably, LMIC residents face double the mortality risk from chronic conditions compared to their high-income counterparts (World Health Organization, 2022), with dementia prevalence projected to more than double by 2050 (Kalaria et al., 2024). Unlike high-income settings with established care systems, most LMICs lack the infrastructure to manage concurrent multimorbidity and cognitive decline (Bloom & Luca, 2016). This crisis is compounded by epidemiological transitions that may be creating cohort-specific vulnerability patterns, where older adults face cumulative disadvantages from untreated conditions, while younger cohorts encounter earlier-onset multimorbidity without adequate care access.

Mexico’s aging population exemplifies the challenges in LMICs, where birth cohorts face divergent health trajectories shaped by historical shifts in education, wealth, and disease patterns. While the proportion of adults aged 60+ will more than double to 25% by 2050 (UN, 2020), newer cohorts present a paradox: though better educated, they also experience a high burden of multimorbidity (Agudelo-Botero et al., 2024; Díaz-Venegas et al., 2019; Gutierrez et al., 2024; Jin et al., 2023; Ma et al., 2024; Rojas-Huerta et al., 2022; Zhou et al., 2024). This contradiction manifests clearly in cognitive health trends: while improved education has boosted cognitive function in recent cohorts (Gutierrez et al., 2024), these gains are increasingly threatened by earlier chronic disease onset. For example, Mexicans aged 60+ in 2015 had 27% higher dementia odds than their same-aged counterparts in 2001, tracking an 8%–9% rise in diabetes and hypertension prevalence (Mejia-Arango et al., 2021). The cardiometabolic disease burden follows a steep age-dependent trajectory: while affecting 27.6% of all Mexican adults (18+ years), prevalence escalates to 44.5% among those aged 60 and over, with women experiencing particularly rapid growth (38.2% increase vs. 31.1% in men between 2001 and 2018; Agudelo-Botero et al., 2024; Rojas-Huerta et al., 2022). This tension between rising educational attainment and high multimorbidity creates competing forces across generations. While education confers cognitive protection, its benefits are increasingly offset by the earlier onset and heavier burden of chronic conditions in younger cohorts (Chowdhury et al., 2023; Zhou et al., 2024). These dynamics underscore the need to investigate how the relationship between multimorbidity and cognitive trajectories has changed across different birth cohorts, particularly in aging populations that have experienced rapid epidemiological and demographic transitions such as in Mexico.

The cumulative disadvantage theory and the concept of cognitive reserve provide insights for understanding how multimorbidity and cognitive decline unfold differently across cohorts with distinct lifelong socioeconomic exposures (Dannefer, 2003; Stern, 2002). While cumulative disadvantage explains how systemic inequalities in education, healthcare, and opportunity compound over time, leading to divergent aging outcomes across socioeconomic groups and birth cohorts (Dannefer, 2003), the concept of cognitive reserve suggests that individuals with higher cognitive reserves (developed through education, occupation, and engaging activities) can better withstand neuropathological damages associated with multimorbidity (Stern, 2002). The cumulative disadvantage theory and cognitive reserve framework together could explain Mexico’s generational differences in aging, particularly due to rapid educational expansion and an epidemiological transition toward chronic diseases (Díaz-Venegas et al., 2019; Downer et al., 2022; Gutierrez et al., 2024; Mejia-Arango et al., 2021). Mexico’s newer cohorts exemplify a health paradox: while expanded education builds cognitive reserve, prolonged exposure to health disparities creates a cumulative disadvantage. This tension means socioeconomic protections against cognitive decline must now counteract both earlier onset and heavier multimorbidity burdens.

Building on this central tension, our study compares two similarly aged (50–60 at baseline) cohorts (Cohort 1: 2001, 2003, 2012; Cohort 2: 2012, 2015, 2021) in Mexico to capture how differences in health and sociodemographic conditions shape cognitive aging across cohorts (Dannefer, 2003; Stern, 2002). Specifically, we examine cohort differences in cognitive decline and assess whether the association of multimorbidity on cognitive function has changed across cohorts. Recent cohorts in Mexico might have experienced cognitive benefits due to higher educational attainment, net worth, and the introduction of universal health coverage between 2003 and 2020 (Downer et al., 2022; Gutierrez et al., 2024; Knaul et al., 2023). However, these improvements might not offset the challenges, such as the negative cognitive association of a high multimorbidity burden (Chowdhury et al., 2023; Mejia-Arango et al., 2021; Rojas-Huerta et al., 2022). Investigating how multimorbidity and cognitive function interact across cohorts with distinct socioeconomic and health compositions provides insights into the health and social needs of aging populations in Mexico and other LMICs facing similar demographic and epidemiological transitions.

## Study Objectives

This study proposes two main hypotheses. First, we expect to observe cohort differences in cognitive decline, specifically cohort 1 (later born, 1941–1951) exhibiting steeper declines in annual average global cognitive function scores (GCFS) after adjusting for sociodemographic characteristics and multimorbidity. Second, we hypothesize that the association between multimorbidity and the annual rate of change in GCFS is moderated by birth cohort, such that the magnitude of the association of multimorbidity on annual declines in GCFS is greater among the more recent cohort (born 1952–1962) compared to the earlier cohort (born 1941–1951) while controlling for SES. By testing these hypotheses, this research aims to advance understanding of how multimorbidity influences cognitive function in Mexico, considering the role of cohort membership and the broader social and epidemiological changes each cohort represents.

## Research Design and Methods

### Data Sources

**Figure 1.**
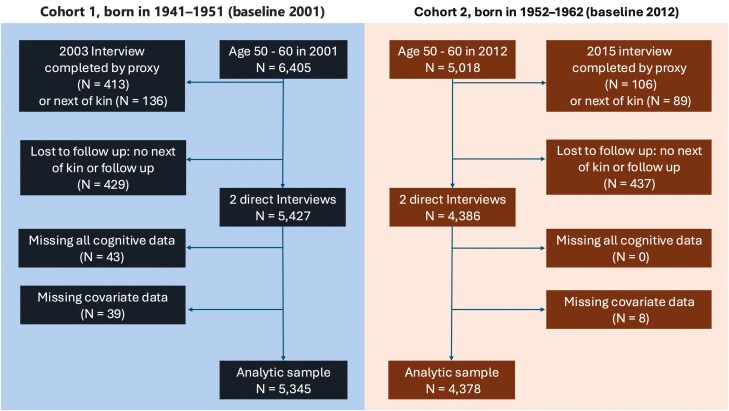
Sample selection across cohorts age 50–60 at baseline (Cohort 1 born 1941–1951: 2001–2012 and Cohort 2 born 1952–1962: 2012–2021).

The MHAS is a national longitudinal study of adults 50 years and older in Mexico. The first wave was collected in 2001, with follow-up interviews in 2003, 2012, 2015, 2018, and 2021. A refresher cohort was added in 2012 (born in 1952–1962; Wong et al., 2017). We used the MHAS datasets with imputed scores to account for missing cognitive data. This includes data from two 10-year birth cohorts (Cohort 1: born 1941–1951, *n* = 6,405; Cohort 2: born 1952–1962, *n* = 5,018) ages 50–60 at baseline across three data time points for each cohort (C1: 2001, 2003, and 2012; C2: 2012, 2015, 2021). Figure 1 shows the selection of the analytical sample. We included respondents who had at least the first two direct interviews with complete cognitive data and no missing covariates (Figure 1). Very few respondents were excluded because they had missing cognitive data (Cohort 1, 2001: *n* = 43) or missing covariates of interest (Cohort 1, 2001: *n* = 39; Cohort 2, 2012: *n* = 8). The final analytic sample with at least two direct interviews and no missing covariates was 5,345 for cohort 1 (2001) and 4,378 for cohort 2 (2012).

### Dependent Variable: Cognitive Function

GCFS are important as they assess cognitive abilities and provide valuable screening and insight into potential cognitive impairments and the cognitive status of individuals. The MHAS cognitive battery measured cognitive function and was administered during the direct interviews (Michaels-Obregón et al., 2020). The MHAS cognitive battery is derived from the Cross-Cultural Cognitive Examination producing an indicator of cognitive function that is preferred for populations with low levels of literacy or education (Glosser et al., 1993; Michaels-Obregón et al., 2014). The cognitive battery includes eight cognitive tasks that each assess different cognitive domains. However, only five tasks were included across all MHAS waves: (a) Verbal learning (respondent listens to eight words and repeats them); (b) Verbal Recall (respondent asked to repeat as many words from the list provided); (c) Visual scanning (measured by asking respondents to circle all figures identical to a specific stimulus shown previously within an array of different stimuli, they are given 60s to do so); (d) Visio constructional (measured by having respondents copy a figure, they have 90s); and (e) Visual Memory or Constructional Praxis recall (through having respondent remember the two figures previously copied; Glosser et al., 1993).

Scores for verbal learning and recall ranged from 0 to 8, visual scanning scores ranged from 0 to 60, and visio constructional and visual memory scores ranged from 0 to 2, for a total of 80 points. Scores increased slightly in subsequent waves; however, the methodology by Michaels-Obregón and colleagues was followed so that scores in all waves ranged from 0 to 80 (Michaels-Obregón et al., 2020). We created a *z*-score for each cognitive measure using the sample mean and standard deviation for the first wave of each cohort as in previous literature (Gutierrez et al., 2024; Torres et al., 2020, 2021). We then averaged the *z*-scores of the assessments to create the composite GCFS. We standardized the composite GCFS using the wave 1 GCFS mean and *SD*.

Similar to previous research using MHAS, we found an unanticipated improvement in cognitive performance between the 2003 and 2012 waves driven by the visio constructional and visual memory assessments (Torres et al., 2020, 2021). Therefore, we used a modified GCFS which includes only three assessments (verbal learning, verbal recall, and visual scanning) as our primary outcome throughout the paper. Additionally, we conducted sensitivity analysis using the full standardized GCFS, which incorporates all five cognitive assessments (verbal learning, verbal recall, visual scanning, visio-constructional, and visual memory), to assess the robustness of our findings. Higher values in both sets of scores indicate better cognitive function, aligning with studies that explore cognitive decline in aging populations (Avila et al., 2020; Camarillo et al., 2023; Dove et al., 2023; Gutierrez et al., 2024; Torres et al., 2020, 2021).

### Main Independent Variable: Multimorbidity

The primary independent variable in this study is average multimorbidity, defined as the presence of two or more chronic health conditions at any point during the study period. Informed by the existing literature and prevalences of cardiometabolic conditions, we include four self-reported physician-diagnosed chronic conditions: diabetes, heart disease, stroke, and hypertension (Dove et al., 2023; Fajardo Dolci et al., 2023; Jin et al., 2023; Khondoker et al., 2023; Souza et al., 2021). Cardiometabolic multimorbidity represents a set of highly prevalent conditions in Mexico that is associated with cognitive decline, affecting both physical and mental health. These conditions were reported from the question “Has a physician ever told you that you have X condition…?” with a response of no (0) or yes (1). These chronic conditions were summed at each wave and the multimorbidity variable was categorized as ever having multimorbidity (2 or more chronic conditions) during the study period (1) or never having multimorbidity (0). This variable is useful for understanding the cumulative burden of cardiometabolic conditions on cognitive outcomes and is expected to show a significant association with cognitive decline in aging cohorts.

### Additional Control Variables

Following previous research (Downer et al., 2022; Gutierrez et al., 2024; Jin et al., 2023) and building on the cumulative disadvantage theory, sociodemographic variables are included as control variables to account for potential confounders in the relationship between multimorbidity and cognitive function. These measures were taken at baseline and grouped into five categories: socioeconomic (education and net worth), sociodemographic (sex and marital status), health status (self-reported health and health insurance), health risk factors (smoking and alcohol), and locality size. Each variable is coded as follows. Education: none (0), 1–5 years corresponding to incomplete elementary (1), 6 years corresponding to complete elementary (2), 7–9 years corresponding to secondary (3), 10+ years corresponding to postsecondary (4). Net worth (in pesos) was constructed by the MHAS team, which considered individual and couple total value of houses, businesses, other properties, capital assets, vehicles, and other debts. We then categorized it into quartiles for each cohort. Sex: male (0), female (1); marital status includes four categories: married or in a civil union (0), separated or divorced (1), widowed (2), or single (3) and was recoded into a binary: being in a union (0) and not being in a union (1). Self-reported health: poor and fair (0), excellent, very good, or good (1); health insurance: no (0), yes (1); current smoking and alcohol consumption was measured as no (0) or yes (1), and locality size: <100,000 people (0) or 100,000+ people (1).

### Statistical Analysis

Descriptive statistics and bivariate tests (chi-square and *t* tests) describe and compare the demographic and health differences across cohorts at baseline, 2001 for Cohort 1 (born 1941–1951) and 2012 for Cohort 2 (born 1952–1962). Bivariate tests were used to examine multimorbidity across cohorts among individuals with data available at each wave (see Appendix): Waves 1 and 2, cohort 1 (2001) *n* = 5,345 and cohort 2 (2012) *n* = 4,378, and wave 3, Cohort 1 (2001) *n* = 4,027 and Cohort 2 (2012) *n* = 3,532.

For the longitudinal analysis, we control for sample selection (attrition) and learned effects of taking the cognitive tests over time. Sample selection is controlled by estimating inverse probability weights to account for biases due to mortality and sample attrition (Weuve et al., 2012). The inverse probability weights were calculated on the predicted survival probability across waves 1 and 2 and between waves 2 and 3 for each cohort (Weuve et al., 2012). We use the inverse of these probabilities as weights in our statistical models. Learned effects are controlled by including an indicator variable representing “the visit 1” to account for learning effects. As suggested by Vivot et al. (2016), “the visit 1” indicator appeared to perform better than two alternatives (number of prior tests and the square root of the number of prior tests) when accounting for learned effects. The indicator variable is coded as 0 for the first interview and 1 for subsequent waves where positive coefficients reflect an improvement in performance following the initial assessment (e.g., 0, 1, 1).

We used a growth curve model controlling for sex, self-reported health, health insurance, locality size, marital status, smoking, alcohol consumption, education, and net worth to examine cohort differences in standardized cognitive function scores. We use age, centered at 55 as it is a meaningful midpoint of our baseline age (50–60). The multilevel model included a random intercept and random slope model centered on age (heretofore called age), allowing each cohort to have a distinct trajectory. The slope parameter in this case represents the average rate of change in the average cognitive score as people age. The model provides for both the estimation of the fixed population parameters (fixed effects) estimated by the growth model of the sample and within individual variability in intraindividual growth model parameters (random effects). The models included all main effects and the interaction between age and cohort to determine the annual change in the cognitive score. Statistical analysis was conducted using SAS 9.4 and R 4.4.1.

The models were specified as follows:


$$\begin{array}{l} GCFS_{ti}=\beta_{0i}+\beta_{1i}\left(Age_{ti}\right)+\beta_{k}Z_{i}+\epsilon_{ti} \end{array}$$

where a person *i*’s modified GCFS at age *t*, *GCFS*_t*i*_, is a function of an individual-specific intercept parameter (β_0*i*_), individual-specific slope (β_1*i*_) that captures the rate of decline or improvement per year (*Age*_*ti*_, time metric), and a residual error term (ϵ_*ti*_).

We modeled the individual specific intercepts (β_0*i*_), and slope (β_1*i*_) as follows:

for the intercept: β_0*i*_ = γ_00_ + *u*_0*i*_ and for the slope: β_1*i*_ = *γ*_10_ + *u*_1*i*_

where *γ*_00_ and *γ*_10_, correspond to the average intercept and slope, and *u*_1*i*_ and *u*_0*i*_ correspond to the random deviations from those means. The full model specification is shown in Appendix A.

In this setting, β_0*i*_ and β_1*i*_ vary across individuals (random effects), and we use this variability to test the two hypotheses; *u*_0i_ and *u*_1i_ represent residual dispersion in β_0*i*_ and β_1*i*_, respectively, after controlling for covariates *Z*_*i*_ and ϵ_it_ is a normally distributed error with homogeneous variance across individuals. For example, to test the first hypothesis we include all main effects and the interaction between age and cohort to assess the annual average change in cognitive scores between the cohorts. We test the second hypothesis by running a model adding a three-way interaction between multimorbidity, cohort, and age. This allows us to assess whether the link between multimorbidity and annual average changes in cognitive scores differs by cohort. We present simplified tables of coefficient estimates (full tables in the Appendix) and produce figures with predictive margins of cognition holding the other covariates at their means to visualize the direction and amount of change throughout the age range of our sample. This allows us to show cohort-specific within-person changes over time in GCFS.

## Results

**Figure 2.**
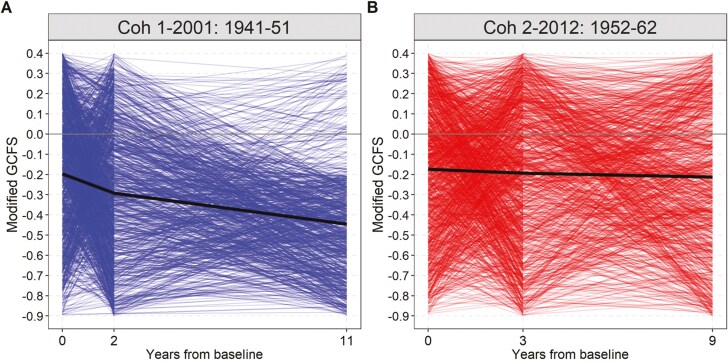
Average modified global cognitive function score (GCFS), thick line, by cohort and years from baseline across waves. (A) Cohort 1, 2001: 1941–1951, (B) Cohort 2, 2012: 1952–1962. *Note*: each thin line represents a respondent’s score in each wave with the thick line representing the average score among respondents in each wave.

Figure 2 presents the average standardized (*z*-score) modified cognitive function scores (heretofore modified GCFS) by cohort across three waves, representing years since baseline, with thin lines representing individual trajectories and thick lines indicating average scores per wave. The figure reveals a consistent annual decline in modified GCFS for both cohorts, with Cohort 1 (2001) experiencing a steeper decline than Cohort 2 (2012). Additional figures in the Appendix (Appendix Figure 1) further disaggregate average *z*-scores by multimorbidity status, showing that respondents with multimorbidity consistently exhibit lower average modified GCFSs (worse cognitive function) and experience a faster decline over the study period, particularly in Cohort 1 (2001). Together, these figures illustrate varying trends in cognitive trajectories across cohorts and by multimorbidity status.

Descriptive results for the covariates are shown in Table 1. Overall, respondents in Cohort 2 (2012) have significantly better background attributes than those in Cohort 1 (2001). For example, Cohort 2 has higher SES (more education and net worth), similar sociodemographic characteristics (except for a higher proportion of females), better health status (e.g., higher fraction of insured individuals and lower fraction of people with risky health behaviors—smoking and drinking) with a slightly lower fraction living in more populous places (100,000+ people). When examining sample characteristics by multimorbidity status across cohorts, we find a similar pattern with respondents in Cohort 2 with multimorbidity having consistently better background characteristics than their counterparts in Cohort 1.

**Table 1.** Baseline Characteristics for Each Cohort and by Multimorbidity Status (Cohort 1 born 1941–1951: 2001–2012 and Cohort 2 born 1952–1962: 2012–2021)

	Cohort 1 (2001)	Cohort 2 (2012)	*p* Value	Multimorbidity		*p* Value
Characteristic				Cohort 1 (2001)	Cohort 2 (2012)	
	*N* = 5,345	*N* = 4,378		*N* = 1,319	*N* = 1,211	
**Socioeconomic (SES)**						
**Education (%)**						
0 years	17. 40	9.16	<.001	18.88	11.40	<.001
1–5 years	33.73	24.05	<.001	36.77	27.00	<.001
6 years	21.68	24.60	<.001	23.35	26.92	.038
7–10 years	15.34	23.50	<.001	12.96	20.31	< .001
10+ years	11.84	18.68	<.001	8.04	14.37	<.001
**Net worth (median)**						
Q1	30,000.00	28,000.00	<.001	34,500.00	29,785.40	<.001
Q2	150,000.00	310,000.00	<.001	152,572.36	300,000.00	<.001
Q3	330,000.00	688,934.88	<.001	319,988.59	629,559.09	<.001
Q4	780,000.00	1,756,281.13	<.001	696,888.88	1,608,189.75	<.001
**Sociodemographic**						
**Sex (%)**						
Female	56.97	63.11	<.001	64.14	69.28	.006
Male	43.03	36.89	<.001	35.86	30.71	.006
**Marital status (%)**						
In a union	77.42	77.25	.843	78.32	78.36	.976
Not in a union	22.58	22.75	.843	21.68	21.64	.976
**Health status**						
**Self-reported health (%)**						
Fair or poor health	59.59	59.25	.735	76.27	75.56	.675
Excellent, very good, or good	40.41	40.75	.735	23.73	24.44	.675
**Health insurance (%)**						
Yes	61.83	86.55	<.001	66.26	90.67	<.001
No	38.17	13.45	<.001	33.74	9.33	<.001
**Health risk factors (%)**						
Current smoker	19.48	15.08	<.001	15.09	11.4	.006
Currently drinks alcohol	35.3	27.43	<.001	28.51	20.97	<.001
**Locality size (%)**						
100,000+ people	61.07	59.95	.047	50.86	45.93	.015
Less than 100,000 people	38.93	40.05	.047	49.14	54.07	.0152

*Note*: Q1, Q2, Q3, and Q4 represent the quartiles of the net worth distribution.

## Results from Multilevel Models

Table 2 presents a summarized set of coefficients estimated from the growth models on annual standardized modified cognitive function scores controlling for sociodemographic variables (a full table of results is shown in the Appendix). Model 1 includes all main effects and the interaction between age and cohort to assess the average annual change in the standardized modified cognitive scores between the cohorts. Results indicate that Cohort 2 (2012) experienced a slower average rate of annual modified cognitive decline compared to Cohort 1 (2001; *β*: 0.02, 95% confidence interval [CI] 0.01–0.02). Model 2 examines whether the association of multimorbidity with cognitive decline differs by cohort. Findings suggest that the association of multimorbidity on annual cognitive decline does not significantly vary by cohort (*β*: 0.001, 95% CI: −0.01 to 0.01), indicating that cohort membership does not moderate the association between multimorbidity and cognitive function. In other words, the association of multimorbidity with cognitive decline is similar between the cohorts.

**Table 2.** Linear Mixed Effect Models for the Association of Multimorbidity on Standardized Modified Cognitive Function Scores Over Time (Cohort 1 born 1941–1951: 2001–2012 and Cohort 2 born 1952–1962: 2012–2021)

Variable	Modified GCFS *β* (95% CI)	*p* Value
Model 1: annual decline (Cohort 2012 * Age)		
Age	−0.02 (−0.03 to −0.02)	<.001
Cohort 2001	Reference	
Cohort 2012 * Age	0.02 (0.01–0.02)	<.001
Model 2: annual decline (Cohort 2012 * Multimorbidity * Age)		
Cohort 2001, no multimorbidity	Reference	
Cohort 2012 with multimorbidity	0.001 (−0.01 to 0.01)	.876

*Notes*: *α* ≤0.05 significance level. All models include a random intercept and slope for age and control for sex, self-reported health, locality, marital status, smoking, alcohol consumption, education, and net worth.

**Figure 3.**
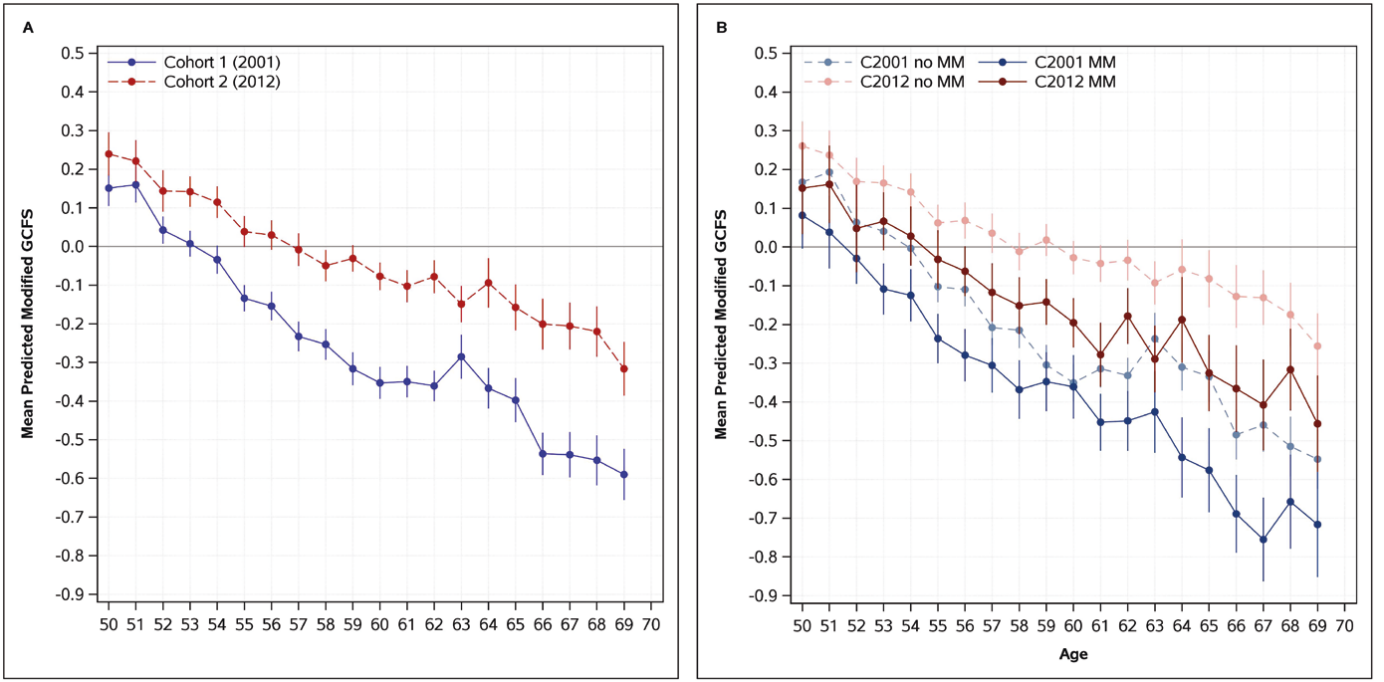
Predicted average modified global cognitive function scores (GCFS) across cohorts (Cohort 1 born 1941–1951: 2001–2012 and Cohort 2 born 1952–1962: 2012–2021) by age and by multimorbidity (none = dashed line, multimorbidity = solid line) from model 1 (A) and model 2 (B) from Table 2. (Cohort 1 born 1941–1951: 2001–2012 and Cohort 2 born 1952–1962: 2012–2021). *Note*: MM stands for multimorbidity.

To illustrate the associations in models 1 and 2 from Table 2, we predicted the average standardized modified cognitive scores by age (Figure 3). Negative scores represent worse cognition relative to baseline. Results suggest that scores decline with age, falling below the baseline average much sooner in Cohort 1 (2001), compared to Cohort 2 (2012). The predicted values from model 1 (Figure 3A) indicate a slower decline by age in Cohort 2 (red) compared to Cohort 1 (blue). These cohort differences are statistically significant across all ages, except at younger ages, with larger differences starting after age 55. In model 2 (Figure 3B), which includes a breakdown by multimorbidity status, the overall age-related patterns remain similar, however, there are no significant cohort differences in the rate of decline. Although Cohort 1 (2001) shows consistently lower scores across all age groups compared to Cohort 2 (2012), both cohorts exhibit a similar rate of decline over time. Individuals with multimorbidity consistently exhibit lower predicted values than those without multimorbidity at each age. Additionally, the gap between individuals with and without multimorbidity widens with age at a comparable rate in both cohorts. These findings indicate that while rates of cognitive decline differ in the overall cohort, the association of multimorbidity with these rates does not differ. In other words, the negative association of multimorbidity on cognitive trajectories remained similar between cohorts.

### Sensitivity Analysis

We also conducted a sensitivity analysis using the standardized full GCFS outcome that includes visuo-constructional and visual memory assessments to the modified score we just described. The Appendix presents the results of this analysis (Appendix: Figures 2–4 and Table 3). The main findings indicate that the cohort trajectories remain similar to those using the modified score with Cohort 1 consistently showing lower average scores (worse cognition) and the association of multimorbidity on the annual rate of decline in average full GCFS showing no significant interaction by birth cohort. However, adding visuo-constructional and visual memory assessments to the modified score leads to an unexpected increase in the full GCFS score between 2003 and 2012, which is consistent with prior findings by Torres et al. (2020, 2021).

## Discussion

We used data from the Mexican Health and Aging Study to compare age trajectories of modified cognitive function scores (only verbal learning, verbal recall, and visual scanning) of Mexican older adults aged 50–60 (at baseline) across two cohorts of similar age who were born ten years apart. Our study has two main findings. First, we found significant cohort differences in age trajectories of average modified cognitive function decline. The results indicate that the recently born cohort, Cohort 2 (2012), experienced a slower rate of average annual decline compared to the later-born Cohort 1 (2001). Second, our analysis of whether the association of multimorbidity with cognitive decline differs by cohort found no statistically significant association. This indicates that the association of multimorbidity with cognitive decline is similar in both cohorts.

Our study reveals a defining paradox of Mexico’s epidemiological transition: recent cohorts of older adults (in 2012) exhibit slower cognitive decline than previous generations (in 2001), despite carrying greater multimorbidity burdens (Appendix Table 1). This pattern reflects two competing forces reshaping cognitive aging trajectories. First, substantial socioeconomic improvements, particularly rising educational attainment and unmeasured healthcare improvements as a result of expanded healthcare access through reforms like Seguro Popular, appear to provide protective benefits, consistent with established links between education and cognitive reserve (Díaz-Venegas et al., 2019; Mejia-Arango et al., 2021). While we found a slower overall decline in cognitive function in recent cohorts, the association of multimorbidity on cognitive trajectories remained similar between cohorts. This dissociation suggests Mexico’s socioeconomic progress may be providing population-level resilience that prevents the cognitive consequences of multimorbidity from becoming more severe among recent cohorts. These findings highlight Mexico’s unique position in global aging trends, where rapid improvements in social determinants of health create differing contexts within each cohort.

Among these factors, educational attainment stands out as it is strongly linked to higher cognitive function in later life (Díaz-Venegas et al., 2019; Mejia-Arango et al., 2021). While testing the association of education and cognition was not our primary focus, the birth cohort design inherently captures underlying improvements in education between the 2001 and 2012 cohorts. Previous literature using MHAS cross-sectional data consistently attributes recent cohorts’ superior cognitive performance to these educational gains (Camarillo et al., 2023; Díaz-Venegas et al., 2019; Gutierrez et al., 2024; Mejia-Arango et al., 2021). For example, research on cohort differences in Mexico indicates that older adults (60+) from more recent cohorts (in 2018) exhibited higher cognition than those from earlier cohorts (in 2001); however, these advantages diminish when accounting for education, underscoring its protective role (Downer et al., 2022; Gutierrez et al., 2024). Our longitudinal approach extends these findings in Mexico by showing that cohort cognitive advantages are also observed when examining cognitive decline. For example, our regression results indicate that respondents with higher educational attainment experienced better average modified cognitive scores than their counterparts with no schooling (see Appendix Table 2). These findings suggest that educational opportunities and other societal improvements may contribute to cognitive reserves in recent cohorts of Mexican older adults (Stern, 2002). While cross-sectional studies have been important for documenting baseline disparities in Mexico, our longitudinal results highlight the need to examine how early-life educational investments shape cognitive functioning as people age (i.e., aging trajectory).

Our findings align with research from high-income countries showing that more recent cohorts tend to have better cognitive function and slower rates of decline, likely driven by socioeconomic improvements (Downer et al., 2019; Guo & Zheng, 2023; Yang et al., 2024). These patterns align with the concept of cognitive reserve, which suggests that individuals from more advantaged cohorts may be better equipped to withstand neuropathological changes that contribute to cognitive decline (Stern, 2002). For example, Yang et al. (2024) examined this topic between 10-year birth cohorts (born 1890–1983) across two decades and found that recent-born cohorts in the United States had small but significantly slower cognitive decline than older cohorts due to advances in educational attainment during the post-WWII period. Similarly, a German study comparing cohorts 20 years apart in the mid-60s found that higher educational attainment among more recent cohorts was linked to improved performance across all cognitive assessments over time (Degen et al., 2022). We also observed improvements in SES (education and net worth) among the recent Cohort 2 (2012) as shown in Table 1, and our results from the regressions (Appendix Table 2) show that education has a significant association with average modified cognitive scores whereby those with higher education experienced slower declines over time. Our future work will explore the role of SES on cohort differences in the annual rate of cognitive decline in greater detail. While SES remains pivotal in cognitive aging, the high burden of multimorbidity poses a challenge to cognitive health in Mexico.

Given the high prevalence of multimorbidity in Mexico, it is essential to assess whether its association with cognitive decline differs across cohorts, particularly as advancements in healthcare and chronic disease management may mitigate some of its detrimental consequences. Recent cohorts face a high burden of multimorbidity, primarily driven by cardiometabolic diseases (such as hypertension, diabetes, and heart disease) which are known to be negatively associated with cognitive function (Dove et al., 2023; Fajardo Dolci et al., 2023; Jin et al., 2023; Khondoker et al., 2023; Souza et al., 2021). However, the extent to which this relationship has changed across cohorts remains unclear. Building on this research gap, our second hypothesis uniquely focused on whether the association of multimorbidity on the rate of change in the modified cognitive scores differed across cohorts. Contrary to our hypothesis, our results suggest that the association of multimorbidity with cognitive decline remained similar across the cohorts. One explanation is that unmeasured improvements in chronic disease management, such as better access to medications and healthcare interventions may have helped mitigate the detrimental association between multimorbidity and cognitive decline among recent cohorts (Camarillo et al., 2023; Knaul et al., 2023). For example, the implementation of Mexico’s Seguro Popular in the early 2000s expanded healthcare access, improving detection and treatment for chronic conditions, which may have contributed to better cognitive health outcomes in more recent cohorts (Knaul et al., 2023). Supporting this idea, Camarillo et al. (2023) found that recent cohorts (in 2018) of older adults (age 60+) with diabetes in Mexico were more likely to treat their condition than those in 2001. These advancements in health insurance coverage and chronic disease management may have played a role in buffering the negative association of multimorbidity on cognitive decline, potentially explaining why we did not observe significant differences in the relationship of multimorbidity on cognitive decline across cohorts (Camarillo et al., 2023). These findings underscore the need for further research on the link between multimorbidity and cognitive function, especially given evolving approaches to chronic disease management and their potential influence on cognitive trajectories across different cohorts.

An important strength of our study is its longitudinal design, which allows us to examine the association of multimorbidity with average annual rate of modified GCFS scores and evaluate differences across cohorts in Mexico. By including two cohorts aged 50–60 at baseline, 11 years apart, our study provides a unique opportunity to assess how cohort-specific differences in health conditions, such as multimorbidity, and sociodemographic factors shape cognitive trajectories among older adults in Mexico. Despite our study’s strengths, we acknowledge some important limitations. For example, our study only focused on three cognitive domains. However, these are the only cognitive assessments included in the first wave of the MHAS, which limits using cognitive assessments added in future waves. Additionally, multimorbidity is self-reported doctor diagnosis which might be subject to some recall bias or underreporting, especially among those who are underinsured. Thus, our measure of multimorbidity does not account for the incidence or severity of chronic conditions that could affect cognitive outcomes. Future studies examining chronic disease incidence and management may provide further insight. Lastly, our study pertains only to cohorts aged 50–60 years at baseline, about 11 years apart. Future studies should also examine whether the findings are replicable with other age groups or cohorts with larger time intervals between cohorts. Despite our limitations, our study leverages rich data from a nationally representative dataset in Mexico (MHAS), which is relevant for addressing emerging health challenges in LMICs, and other countries with a high prevalence of multimorbidity.

## Supplementary Material

gnaf143_suppl_Supplementary_Materials

## Data Availability

This study uses data from the Mexican Health and Aging Study (MHAS), a publicly available longitudinal survey (available at mhasweb.org). Researchers can access the MHAS data, including survey waves and documentation, at www.mhasweb.org upon registration. The Appendix shows the analytic methods and materials used in this study. This study was not preregistered.
