# Supplementary material for: Cohort Changes in Cognitive Function Among Mexican Older Adults from 2001 to 2021
Source: Gerontologist. 2025 May 28;65(7):gnaf143. doi: 10.1093/geront/gnaf143 (PMC12284392; doi:10.1093/geront/gnaf143)
Supplement: gnaf143_suppl_Supplementary_Materials [file gnaf143_suppl_supplementary_materials.docx]

**Appendix (Supplementary information)**

Appendix Figure 1. Average modified global cognitive function score (GCFS), thick line, by cohort; (A) Cohort 1, 2001: 1941-51, (B) Cohort 2, 2012: 1952-62, multimorbidity status, and years from baseline at each wave. The modified score includes only three questions (see text for their definition).
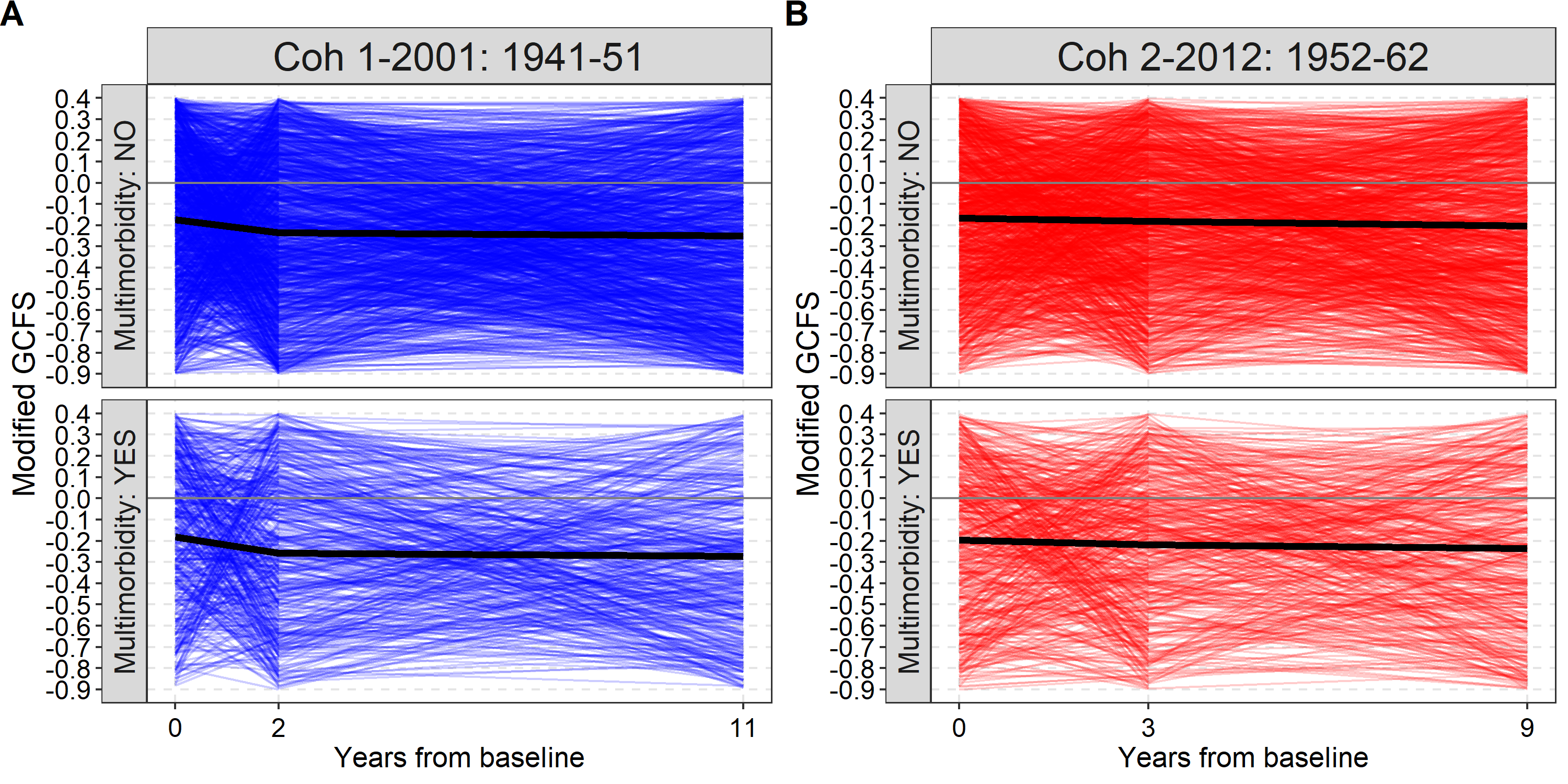


Alt text: Average full GCFS scores for two cohorts, with thick lines highlighting average trends by years from baseline at each wave: Cohort 1 (2001–2012) on the left and Cohort 2 (2012–2021) on the right.

Note: each thin line represents a respondent’s score in each wave with the thick line representing the average score among respondents in each wave.

Appendix Figure 2. Average full global cognitive function score (GCFS), thick line, by cohort (A) Cohort 1, 2001: 1941-51, (B) Cohort 2, 2012: 1952-62, multimorbidity status, and years from baseline at each wave. The full score includes all five questions (see text for their definition). Average full GCFS by multimorbidity status in


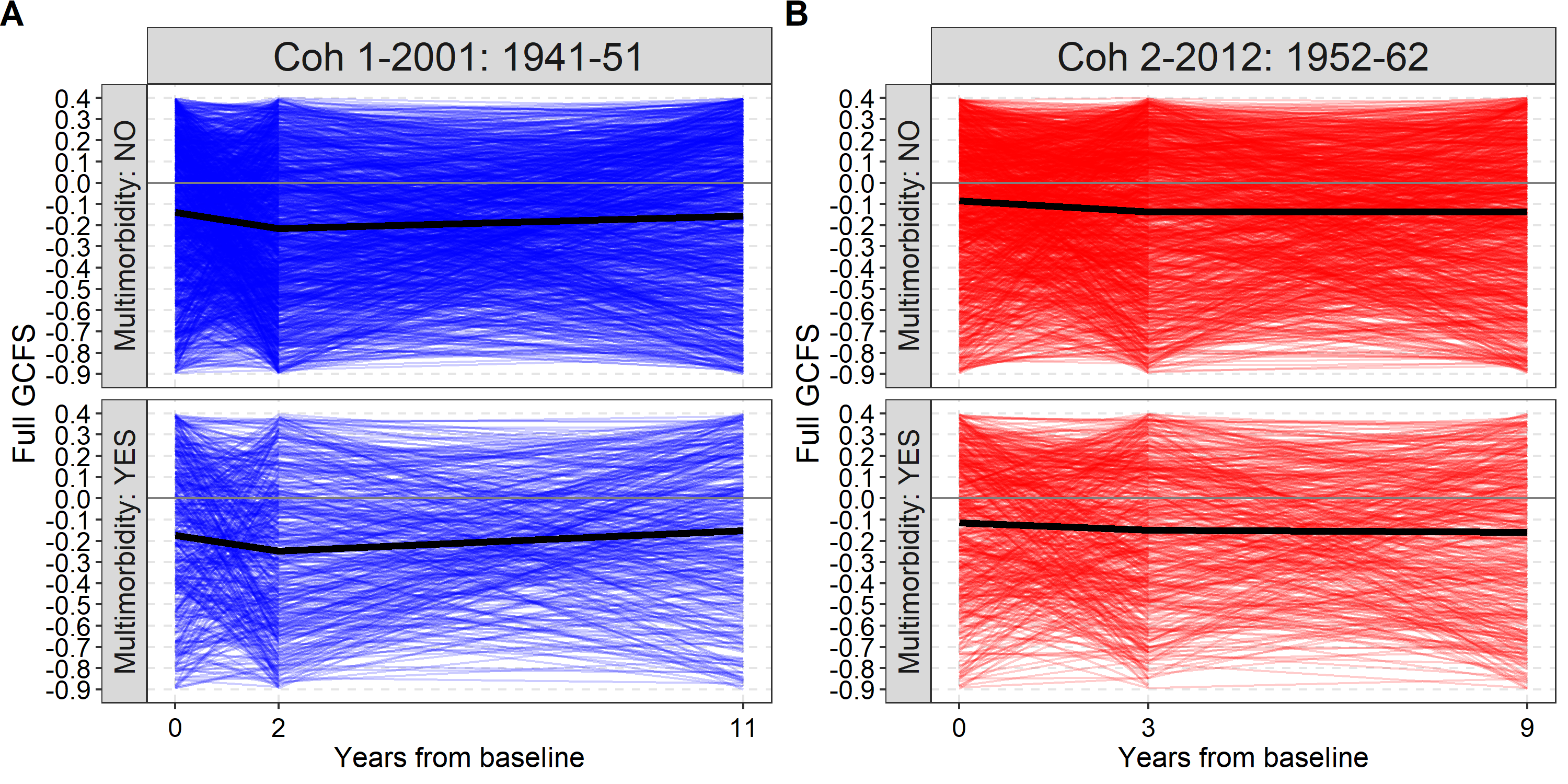


Alt text: Average modified GCFS scores for two cohorts, with thick lines highlighting average trends by years from baseline at each wave: Cohort 1 (2001–2012) on the left and Cohort 2 (2012–2021) on the right.

Note: each thin line represents a respondent’s score in each wave with the thick line representing the average score among respondents in each wave.

Appendix Figure 3. Average full global cognitive function score (GCFS), thick line, by cohort and years from baseline at each wave . The full score includes all five questions (see text for their definition). (A) Cohort 1, 2001: 1941-51, (B) Cohort 2, 2012: 1952-62.


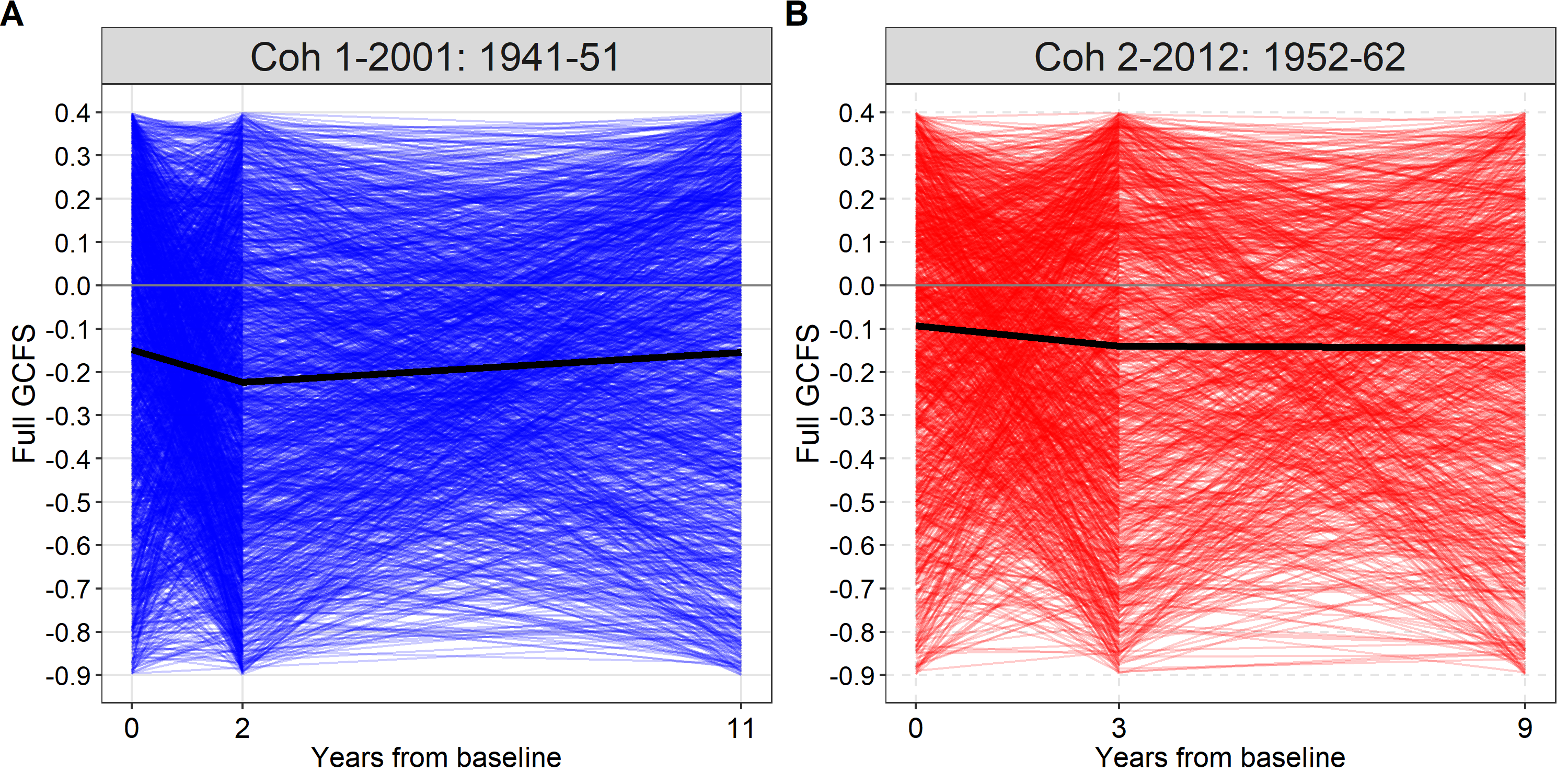


Alt text: Average full GCFS scores for two cohorts, with thick lines highlighting average trends by years from baseline at each wave: Cohort 1 (2001–2012) on the left and Cohort 2 (2012–2021) on the right.

Note: each thin line represents a respondent’s score in each wave with the thick line representing the average score among respondents in each wave.

Appendix Figure 4. Predicted average full global cognitive function scores (GCFS) across cohorts (Cohort 1, 2001 in blue; Cohort 2, 2012 in red) by age and by multimorbidity from model 1 (A) and model 2 (B) from Table 2. (Cohort 1 born 1941-1951: 2001 to 2012 and Cohort 2 born 1952-1962: 2012 to 2021). Note: MM stands for multimorbidity.


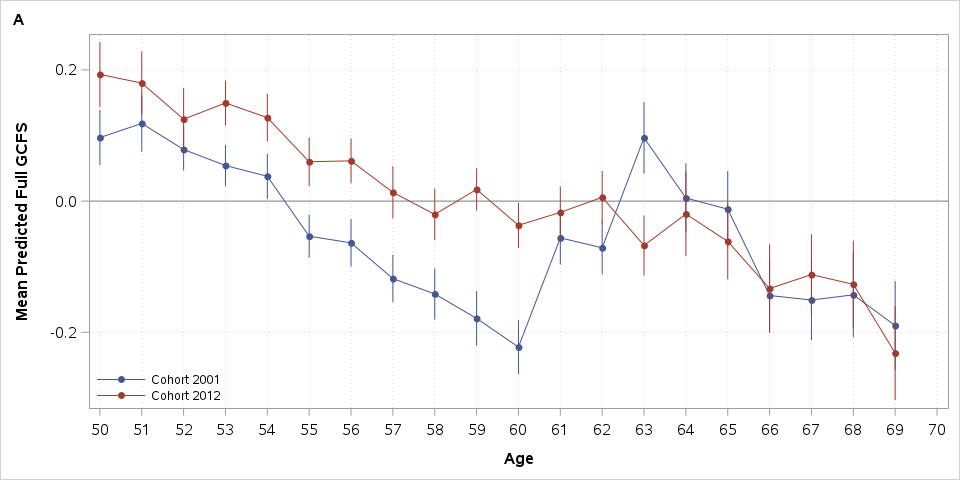


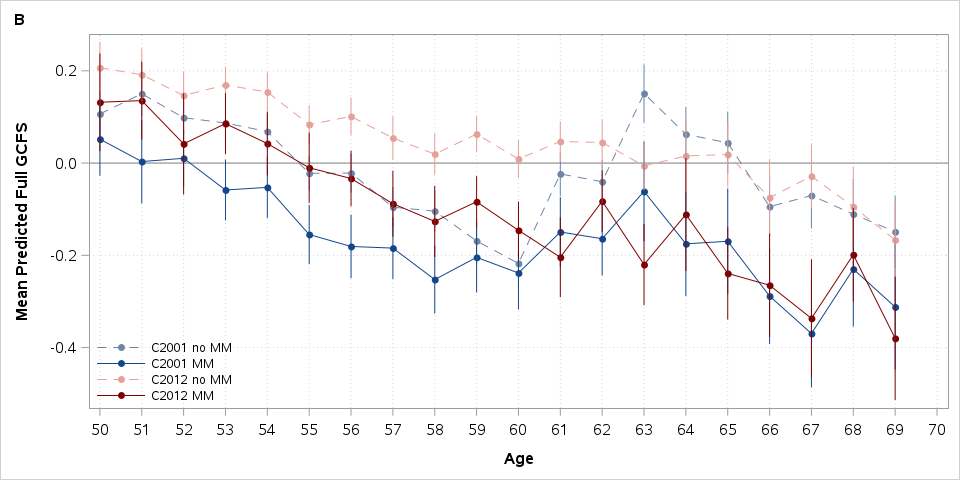
Alt text: Shows predicted average full GCFS scores by age and multimorbidity status (none = dashed line, multimorbidity = solid line) for two cohorts: Cohort 1 (2001–2012, blue) and Cohort 2 (2012–2021, red), shown for Model 1 (panel A) and Model 2 (Panel B).

Appendix Table 1. Comparison of multimorbidity prevalence between the cohorts by wave. Cohort 1 (born 1941-1951) includes participants observed across three waves: wave 1 (2001), wave 2 (2003), and wave 3 (2012). Cohort 2 (born 1952-1962) was similarly observed across three waves: wave 1 (2012), wave 2 (2015) and wave 3 (2021).

|  | Cohort 1 (N = 5,345) | Cohort 2 (N= 4,378) | P Value |
| --- | --- | --- | --- |
| Multimorbidity  Wave 1 | 9.54 | 13.22 | <.001 |
| Wave 2 | 15.27 | 17.36 | 0.005 |
| Wave 3 | 21.16 | 22.96 | 0.05 |
| Ever Multimorbidity | 24.68 | 27.66 | <.001 |

Note: Differences in Chi-square and T-tests between waves are at α ≤ 0.05. Sample size at wave 3, Cohort 1 (n = 4,027), Cohort 2 (n = 3,532).

Appendix Table 2. Linear mixed effect models for **modified** standardized global cognitive function scores (GCFS), age centered at 55 years old.

| Variable | Model 1  Modified GCFS Coefficient | Model 2  Modified GCFS Coefficient |
| --- | --- | --- |
| Intercept | -1.03 (-1.09 to –0.98)*** | -1.09 (-1.15 to -1.03)*** |
| Cohort 2 (2012) x Age | 0.02 (0.01 to 0.02)*** | 0.02 (0.01 to 0.03)*** |
| Cohort 2 (2012) x Multimorbidity x Age |  | 0.001 (-0.01 to 0.01) |
| Cohort 2 (2012) | -0.06 (-0.09 to -0.03)*** | -0.06 (-0.09 to 0.03)** |
| (ref = cohort 1 (2001)) |  |  |
| Age | -0.02 (-0.03 to -0.02)*** | -0.03 (-0.04 to -0.03)*** |
| Ever had multimorbidity  (ref= never had multimorbidity) | -0.07 (-0.10 to -0.04)*** | 0.05 (0.02 to 0.08)** |
| Multimorbidity x Age |  | 0.01 (0.003 to 0.01)*** |
| Socioeconomic status (SES) |  |  |
| Net assets (Ref Q1) |  |  |
| Q2 | 0.03 (-0.01 to 0.06) | 0.03 (-0.01 to 0.06) |
| Q3 | 0.11 (0.07 to 0.14)*** | 0.11 (0.07 to 0.14) *** |
| Q4 | 0.13 (0.10 to 0.17)*** | 0.13 (0.10 to 0.17)*** |
| Years of education (reference: 0 years) |  |  |
| 1-5 | 0.39 (0.35 to 0.43)*** | 0.39 (0.35 to 0.43) *** |
| 6 | 0.71 (0.67 to 0.75)*** | 0.71 (0.67 to 0.75) *** |
| 7-9 | 1.06 (1.02 to 1.11)*** | 1.06 (1.02 to 1.10) *** |
| 10+ | 1.34 (1.27 to 1.37)*** | 1.34 (1.29 to 1.39) *** |
| Sociodemographic |  |  |
| Sex |  |  |
| Females (ref: males) | 0.23 (0.20 to 0.26)*** | 0.23 (0.21 to 0.26)*** |
| Marital Status |  |  |
| In a Union (ref= no union) | 0.03 (-0.001 to 0.06) | 0.03 (-0.001 to 0.06) |
| Self-reported health |  |  |
| Excellent, very good, or good  (ref= fair or poor) | 0.06 (0.03 to 0.08)*** | 0.06 (0.03 to 0.08)*** |
| Health status |  |  |
| Has health insurance (ref= no) | 0.09 (0.06 to 0.12)*** | 0.09 (0.06 to 0.12)*** |
| Current drinker  (ref= not current drinker) | 0.03 (0.004 to 0.06)* | 0.03 (0.004 to 0.06)* |
| Current smoker  (ref= not current smoker | -0.01 (-0.04 to 0.03) | -0.01 (-0.04 to 0.03) |
| Locality size |  |  |
| 100,000+ people  (ref = less than 100,000 people) | 0.12 (0.10 to 0.15)*** | 0.12 (0.10 to 0.15)*** |
| Indicator | -0.13 (-0.15 to –0.11)*** | -0.13 (-0.15 to –0.11)*** |
| Fit (N=9,723) |  |  |
| -2 Log Likelihood | 62,771.8 | 62,780.3 |
| AIC | 62,779.8 | 62,780.3 |
| BIC | 62,808.6 | 62,809.0 |

***p-value <.001, ** p-value <.01, * p-value < .05

Appendix Table 3. Linear mixed effect models on the **full** standardized global cognitive function scores (GCFS) over time, age centered at 55 years old (Cohort 1 born 1941-1951: 2001 to 2012 and Cohort 2 born 1952-1962: 2012 to 2021).

| Variable | Model 1  Full GCFS Coefficient | Model 2  Full GCFS Coefficient |
| --- | --- | --- |
| Intercept | -1.01 (-1.0 to -0.98)*** | -1.02 (-1.07 to -0.97) *** |
| Cohort 2 (2012) x Age | -0.003 (-0.01 to -0.00003) | -0.003 (-0.01 to 0.001) |
| Cohort 2 (2012) x Multimorbidity x Age |  | -0.001 (-0.01 to 0.01) |
| Cohort 2 (2012) | -0.09 (-0.12 to -0.07)*** | -0.09 (-0.12 to -0.07)*** |
| (ref = cohort 1 (2001)) |  |  |
| Age | 0.001 (-0.001 to 0.003) | 0.003 (0.001 to 0.01)** |
| Ever had multimorbidity  (ref= never had multimorbidity) | -0.06 (-0.08 to -0.03)*** | -0.04 (-0.07 to -0.01)** |
| Multimorbidity x Age |  | -0.001 (-0.01 to -0.003)*** |
| Socioeconomic status (SES) |  |  |
| Net assets (Ref Q1) |  |  |
| Q2 | 0.05 (0.01 to 0.08)** | 0.05 (0.01 to 0.08)** |
| Q3 | 0.11 (0.07 to 0.14)*** | 0.10 (0.07 to 0.14) *** |
| Q4 | 0.14 (0.10 to 0.17)*** | 0.14 (0.10 to 0.17)*** |
| Years of education (reference: 0 years) |  |  |
| 1-5 | 0.54 (0.50 to 0.57)*** | 0.54 (0.50 to 0.57)*** |
| 6 | 0.84 ( 0.80 to 0.88)*** | 0.84 ( 0.80 to 0.88)*** |
| 7-9 | 1.13 (1.08 to 1.17)*** | 1.13 (1.08 to 1.17)*** |
| 10+ | 1.34 (1.29 to 1.39)*** | 1.34 (1.30 to 1.39)*** |
| Sociodemographic |  |  |
| Sex |  |  |
| Females (ref: males) | 0.11 (0.08 to 0.13)*** | 0.11 (0.08 to 0.13)*** |
| Marital Status |  |  |
| In a Union (ref= no union) | 0.04 (0.01 to 0.07)** | 0.04 (0.01 to 0.07)** |
| Self-reported health |  |  |
| Excellent, very good, or good  (ref= fair or poor) | 0.05 (0.02 to 0.07)*** | 0.05 (0.02 to 0.07)*** |
| Health status |  |  |
| Has health insurance (ref= no) | 0.09 (0.06 to 0.11)*** | 0.09 (0.06 to 0.11)*** |
| Current drinker  (ref= not current drinker) | 0.03 (0.003 to 0.06)* | 0.03 (0.003 to 0.06)* |
| Current smoker  (ref= not current smoker | -0.01 (-0.04 to 0.02) | -0.01 (-0.04 to 0.02) |
| Locality size |  |  |
| 100,000+ people  (ref = less than 100,000 people) | 0.10 (0.07 to 0.12)*** | 0.10 (0.07 to 0.12)*** |
| Indicator | -0.05 (-0.07 to –0.03)*** | -0.05 (-0.07 to –0.03)*** |
| Fit (N=9,723) |  |  |
| -2 Log Likelihood | 57,850.2 | 57,846.5 |
| AIC | 57,858.2 | 57,854.5 |
| BIC | 57,886.9 | 57,883.2 |

***p-value <.001, ** p-value <.01, * p-value < .05

**Appendix A**

**Modeling approach:**

Equations 1 and 2 represent the model specifications. In the level 1 model, the growth trajectory in global cognitive function score (GCFS) is a function of age.

**Level 1 model (growth trajectory within individuals)**

1. *GCFS*_t_*_i_* = 𝛽_0_*_i_* + 𝛽_1_*_i_* (*Age_ti_* ) + ϵ*_ti_*

where a person i’s global cognitive function score at age *t*, *GCFS*_t_*_i,_* is a function of an individual-specific intercept parameter (𝛽_0_*_i_*), individual-specific slope (𝛽_1_*_i_*) that captures the rate of decline or improvement per year (*Age_ti_*, time metric), and a residual error term (ϵ*_ti_*).

We modeled the individual specific intercepts (𝛽_0_*_i_*), and slope (𝛽_1_*_i_*) as follows:

For the intercept: 𝛽*_0i_* = γ_00_ + u*_0i_*

For the slope: 𝛽*_1i_* = γ_10_ + u*_1i_*

Where γ_00_ and γ_10,_ correspond to the average intercept and slope, and u*_1i_* and u*_0i_* correspond to the random deviations from those means.

To investigate how variability in trajectories was influenced by birth cohort and multimorbidity, we included cohort membership (C1, 2001 vs. C2, 2012) and multimorbidity (yes/no) as a Level 2 predictor in the growth model. We also included time invariant covariates at baseline associated with each individual (𝛽*_k_*Z*_i_*) which include marital status, health insurance, sex, education, net worth, locality size, smoking, alcohol consumption, and self-reported health.

For the intercept: 𝛽_0i_ = γ_00_ + γ_01_cohort*_j_* + γ_02_multimorbidity*_i_* + 𝛽*_k_*Z*_i_*  + u*_0i_*

For the slope: 𝛽_1i_ = γ_10_ + γ_11_ cohort *_j_* _+_ u*_1i_*

The final model is given by: *GCFS*_t_*_i_* = γ_00_ + γ_01_cohort*_j_* + γ_02_multimorbidity*_i_* + 𝛽*_k_*Z*_i_*  + u*_0i_* +

(γ_10_ + γ_11_ cohort *_j +_* u*_1i_*)* *Age_ti_* + ϵ*_ti_*

Which simplifies as: *GCFS*_t_*_i_* = γ_00_ + γ_01_cohort*_j_* + γ_02_multimorbidity*_i_* + 𝛽*_k_*Z*_i_*  + u*_0i_* +

γ_10_ *Age_ti_* + γ_11_ cohort *_j_ Age_ti +_* u*_1i_Age_ti_*  + ϵ*_ti_*

γ_00 :_ represents the mean intercept of GCFS for the reference cohort 1 (2001) without multimorbidity at baseline.

γ_01 :_ represents the mean difference in GCFS between cohorts without multimorbidity at baseline.

γ_02 :_ represents the mean difference in the GCFS between those with multimorbidity and no multimorbidity in Cohort 1(2001) at baseline.

γ_10 :_ represents the mean annual rate of change of GCFS for the reference cohort 1 (2001) with no multimorbidity.

γ_11 :_ represents the mean difference in the annual rate of change in GCFS between cohorts 1 (2001) and 2 (2012). This coefficient captures cohort differences in the annual rate of cognitive change, independent of multimorbidity status.

u*_0i_* and u*_1i_*: represent residual dispersion in 𝛽_0i_ and 𝛽_1i_ after controlling for all covariates in the model.

To test the second hypothesis and examine whether the link between multimorbidity on cognitive function differed between cohorts we modified the growth model by incorporating an interaction between cohort and multimorbidity (γ_13 *_ cohort_j *_ multimorbidity_i_) which represents the link between multimorbidity on cognitive function for individuals in Cohort 2 (2012) compared to Cohort 1(2001).

Level 2

For the intercept: 𝛽_0i_ = γ_00_ + γ_01_cohort*_j_* + γ_02_multimorbidity*_i_* + 𝛽*_k_*Z*_i_*  + u*_0i_*

For the slope:

𝛽_1i_ = γ_10_ + γ_11_ cohort _j_ + γ_12_multimorbidity_i_ + γ_13_ cohort_j_ multimorbidity_i_ + u*_1i_*

The final model is given by: *GCFS*_t_*_i_* = 𝛽_0i_ = γ_00_ + γ_01_cohort*_j_* + γ_02_multimorbidity*_i_* + 𝛽*_k_*Z*_i_*  + u*_0i_*

+ (γ_10_ + γ_11_ cohort _j_ + γ_12_multimorbidity_i_ + γ_13_ cohort_j_ multimorbidity_i_ *_+_* u*_1i_*)*Age*_ti_* + ϵ*_ti_*

Which simplifies as: *GCFS*_t_*_i_* = 𝛽_0i_ = γ_00_ + γ_01_cohort*_j_* + γ_02_multimorbidity*_i_* + 𝛽*_k_*Z*_i_*  + u*_0i_* + γ_10_ Age*_ti_* + γ_11_ cohort *_j_* Age*_ti_* + γ_12_multimorbidity_i_ Age*_ti_* + γ_13_ cohort_j_ multimorbidity_i_ Age_ti_ *_+_* u*_1i_*Age*_ti_* + ϵ*_ti_*

γ12 : represents the mean difference in the link between multimorbidity and the rate of annual change of GCFS between cohorts. This coefficient estimates whether the link between multimorbidity and the annual rate of cognitive function change differs by cohort.

γ13 : represents the mean difference in the link between multimorbidity and the rate of annual GCFS change between cohort 1 (2001) and cohort 2 (2012). This coefficient estimates whether the link between multimorbidity and the annual rate of cognitive change differs by cohort.
